# Fasting reduces the incidence of vincristine‐associated adverse events in dogs

**DOI:** 10.1111/vco.12638

**Published:** 2020-08-26

**Authors:** Margaret E. Duckett, Kaitlin M. Curran, Haley J. Leeper, Carl E. Ruby, Shay Bracha

**Affiliations:** ^1^ Department of Clinical Sciences Carlson College of Veterinary Medicine, Oregon State University Corvallis Oregon USA; ^2^ Veterinary Medicine & Biomedical Sciences Texas A&M University College Station Texas USA

**Keywords:** cancer, chemotherapy, dogs, fasting, toxicity, vincristine

## Abstract

Fasting has been shown to decrease chemotherapy‐associated adverse events (AEs), in part through insulin‐like growth factor (IGF‐1) reduction, and may induce a protective effect on normal cells during chemotherapy treatment in mice and people. The purpose of this study was to evaluate the effect of fasting on constitutional, bone marrow and gastrointestinal (GI) AEs, and serum glucose, IGF‐1 and insulin levels in dogs receiving vincristine. The study was a prospective, crossover clinical trial in tumour‐bearing dogs. Dogs were randomized to be fasted for 24 to 28 hours prior to and 6 hours following their first or second vincristine treatment, and fed normally for the alternate dose. A significant reduction in nausea, anorexia, lethargy and serum insulin was observed when dogs were fasted; however, no significant differences were found in other GI symptoms, neutrophil count, serum glucose or IGF‐1. Fasting prior to vincristine therapy is a safe and effective treatment modality that helped mitigate constitutional and GI AEs in tumour‐bearing dogs.

## 1 INTRODUCTION

Cancer has emerged as the greatest health concern of dog owners, and maintaining quality of life (QOL) is paramount when pet owners are considering treatment options, especially chemotherapy.^1^, ^2^, ^3^ The balance between toxicity, tumour response and patient QOL necessitates the continued investigation of methods to control chemotherapy‐associated toxicity. In the absence of such options, owners may decline chemotherapy‐based treatment for their pets or limit the extent and aggressiveness of these life‐extending treatments.

Chemotherapy agents may cause mild to severe gastrointestinal (GI) side effects, and effective means to mitigate these effects remains elusive.^4^ Vincristine is a commonly used chemotherapeutic drug with adverse events (AEs) such as lethargy, vomiting, diarrhoea or cytopenias can occurring in nearly 70% of treated dogs; however, the majority of these AEs are low grade.^5^ In a retrospective analysis of owner‐reported concerns during chemotherapy, concerns were identified at 48% of appointments, and were significantly related to diarrhoea and remission status.^2^

Another study where owners assessed their dogs' QOL during chemotherapy treatment for lymphoma, the majority of owners felt their dogs QOL remained high and 80% would pursue chemotherapy treatment again. However, 32% felt QOL was acceptable but poorer than before the lymphoma diagnosis.^3^ Due in part to these QOL concerns and potential adverse effects of chemotherapy, anti‐emetic and anti‐diarrheal treatments are often prescribed. One of these treatments, maropitant citrate a selective neurokinin‐1 receptor antagonist, has been shown in a randomized, double‐blinded, placebo‐controlled study to be an effective anti‐emetic in canine cancer patients treated with doxorubicin.^6^ However, a recent randomized, prospective clinical trial evaluating the use of maropitant for GI toxicity associated with vincristine and cyclophosphamide revealed no difference in vomiting, diarrhoea, anorexia or lethargy.^5^

Further attempts to reduce the incidence of chemotherapy related toxicity have been made. In one study, canine patients fasted prior to doxorubicin administration experienced a reduced incidence of vomiting (10% vs 67% when fed normally).^7^ Similarly, a case series of 10 people who voluntarily fasted prior to and after a chemotherapy cycle noted fewer chemotherapy‐related toxicities compared with cycles in which they did not fast.^8^ Fasting cycles have been shown in in vitro and in vivo models to provide both increased malignant cell apoptosis and protective benefits to normal cells when exposed to chemotherapy.^9^, ^10^, ^11^, ^12^, ^13^, ^14^, ^15^ Furthermore, Lee et al showed 60% of tumour bearing mice treated with doxorubicin after fasting achieved long‐term survival while the entire control group died of metastasis or chemotherapy related toxicities.^10^ A recent study in breast cancer patients found a significant reduction in haematological AE when patients fasted prior to treatment.^16^ It is proposed that the protective benefits of fasting on normal cells, including GI and haematopoietic cells, are in part because of reduced levels of insulin‐like growth factor (IGF‐1) and glucose.^10^, ^17^

The purpose of this study was to evaluate the effect of short‐term fasting on incidence and severity of GI, constitutional and haematological AEs in dogs treated with vincristine, as well as to identify any correlative effect of fasting on serum glucose, insulin and IGF‐1 concentration. Thus, we hypothesized that short‐term fasting prior to administration of vincristine would significantly reduce AE associated with chemotherapy, and would reduce serum glucose, insulin and IGF‐1.

## 2 MATERIALS AND METHODS

### 2.1 Patient selection

Client‐owned dogs presenting to Oregon State University Teaching Hospital between June 2016 and April 2018 with the intent of pursuing at least two doses of vincristine (Hospira, Lake Forest, Illinois) as part of their chemotherapy protocol were eligible for enrolment. For dogs receiving a multi‐agent protocol, only AE associated with vincristine were evaluated. Prior to enrolment, dogs diagnosed with lymphoma were permitted to begin treatment with prednisone (0.5‐1 mg/kg PO q24, PAR Pharmaceutical, Chestnut Ridge, New York), cyclophosphamide (250 mg/m^2^ PO rounded to nearest 25 mg capsule (Cipla, Sunrise, Florida ) and furosemide (1‐2 mg/kg PO Vetone, Boise, Idaho). Dogs that experienced GI, constitutive or haematological AE following this treatment were not eligible for enrolment. Dogs on concurrent medications at the time of diagnosis with the potential to alter GI toxicities such as prednisone or a non‐steroidal anti‐inflammatory were excluded unless they had been on that medication for greater than 1 week with no reported GI AEs and were anticipated to be on the medication throughout the study period. Dogs on probiotics were excluded.

All dogs had a physical examination, weight, complete blood cell count (CBC), and serum biochemistry performed prior to enrolment. Dogs were excluded if they weighed <5 kg, had significant alterations on screening blood work suggestive renal or hepatic disease, had a concurrent metabolic disease, pre‐existing chronic GI disease or were clinically suspected to have neoplastic GI involvement. An abdominal ultrasound was not a requirement to be included in the study.

Dogs were required to be fed twice daily or ad lib as part of their normal husbandry, and owners were required to feed a consistent diet during the study period. Dogs were excluded if they had previously been treated with vincristine, were positive for the multi‐drug resistant gene (*MDR‐1*, *ABCB1*) mutation, were typically fed once per day, had a diet change within 1 week of enrolment, or showed symptoms of nausea, vomiting or diarrhoea within 2 days prior to trial enrolment.

The study design, treatment protocol and informed client consent was approved by the Institutional Animal Care and Use Committee. Once the study was complete, dogs were free to pursue further therapy as desired by their owner.

### 2.2 Treatment protocol

A prospective, controlled, crossover clinical trial was implemented to evaluate the effect of short‐term fasting on AE's from chemotherapy, and serum glucose, insulin and IGF‐1. Each dog served as its own control with a crossover design. Randomization was performed via a coin flip. Dogs were fasted prior to their first chemotherapy treatment and then fed their regular feeding routine (twice daily, timing varied with normal husbandry habits) prior to their second chemotherapy treatment or were fed their regular feeding routine prior to their first chemotherapy treatment and fasted prior to their second treatment. Fasted dogs received no food after 12 pm the day prior to the chemotherapy treatment and had free access to water. All dogs were fed normally the evening of their chemotherapy appointment (approximately 6 pm), thus total fasting times ranged from approximately 30 hours for ad libitum fed dogs to 34 hours for dogs fed twice daily. All dogs were evaluated by physical exam, owner assessment of QOL survey and a CBC within 48 hours of each chemotherapy treatment.^18^ Prophylactic anti‐emetic (ie, maropitant, ondansetron and metoclopramide) or anti‐diarrheal (ie, metronidazole and tylosin) medications were not permitted.

Dogs were treated within 1 hour of noon the day of their chemotherapy treatment. Dogs were treated with a standard chemotherapy dosage calculated by body surface area. Vincristine treatments were administered at 0.6 mg/m^2^ intravenously (IV) and given at least 1 week apart. A CBC was scheduled 7 to 10 days after each dose of vincristine. A physical exam and owner assessment QOL survey were performed at each appointment.

### 2.3 Response and toxicity assessment

Clients were sent home with AE journals after each chemotherapy appointment. They were instructed to note any changes in appetite or activity level in the journal as well as any episodes of vomiting, diarrhoea or nausea. GI and constitutional AE were graded based on owner journal documentation and patient history gathered at each appointment according to the Veterinary Cooperative Oncology Group‐Common Terminology Criteria for Adverse Events (VCOG‐CTAE) v1.1 scheme.^19^ Similarly, haematologic AE were graded based on follow‐up CBC data, 7 to 10 days post each vincristine dose. Grading was performed by the attending clinician.

Dogs experiencing grade II or higher vomiting or nausea (according to the VCOG‐CTCAE v1.1) could be treated as clinically indicated with ondansetron and/or maropitant, depending on clinician preference. The same anti‐emetic drug(s) was required to be used as indicated for each dog for the duration of the study period. Dogs experiencing grade II or higher diarrhoea (VCOG‐CTACE v1.1) were treated with metronidazole as indicated based on clinician preference.

Dogs were removed from the study if a grade III AE occurred that precluded continuation of chemotherapy at the same dose, progressive disease was noted, or an owner requested study withdrawal for any reason.

### 2.4 Serum glucose, insulin, and IGF‐1 measurement

Peripheral blood samples were obtained via jugular venipuncture immediately prior to each chemotherapy treatment to attain a fasted and fed sample (total of two time points) for each patient. Glucose was tested through Oregon State University Veterinary Diagnostic Lab. Glucose samples were processed (centrifuged and serum separated) within 1 hour of collection. Samples intended for insulin and IGF‐1 measurement were allowed to clot and the serum was separated by centrifugation. The serum was promptly frozen and stored at −80°C until all samples were collected within the study period. Serum insulin and IGF‐1 concentrations were batched and measured using ALPCO Diagnostics (Salem, New Hampshire) porcine/canine insulin enzyme‐linked immunosorbent assay (ELISA) and IGF‐1 ELISA, respectively.

### 2.5 Statistical analysis

A power analysis was performed to estimate the number of dogs needed to detect a statistically significant difference in AE incidence between fasting and fed time points. Based on the literature, expectations were that dogs would have an AE incidence rate of 10% vs 40% to 60%.^5^, ^7^, ^20^ Using an alpha level of 0.05 and a power of 0.80, a minimum of 13 dogs, each with a fed and fasted time point were needed for enrolment to detect a significant difference in AE incidence. A second power analysis was performed to estimate the number of dogs needed to detect a statistically significant difference in neutrophil count between fasted and fed time points (2500 vs 2000 cells/uL, SD of 300, alpha of 0.05, power of 0.80 calculated minimum of six enrolled dogs with two time points each). Statistical analysis consisted of exact McNemar's tests to evaluate the effect of fasting/feeding and order of treatment on the presence of vomiting, diarrhoea and thrombocytopenia. The Wilcox‐signed rank tests were used to evaluate paired data on GI AE severity. Paired *t* tests were used to evaluate the effect of fasting and order of treatment on neutrophil count, and glucose concentration. Effects of fasting/feeding and order on glucose, insulin and IGF‐1 levels were tested with a linear mixed model to account for within dog correlation of duplicates. The first arm (ie, order = 1) of the study was analysed separately for treatment effects with two parallel groups to evaluate treatment effects in the first treatment only, absent any potential order effects. A *P*‐value of <.05 was considered significant.

## 3 CELL LINE VALIDATION STATEMENT

This study did not involve cell lines; therefore, cell line validation work was not carried out.

## 4 RESULTS

**FIGURE 1:**
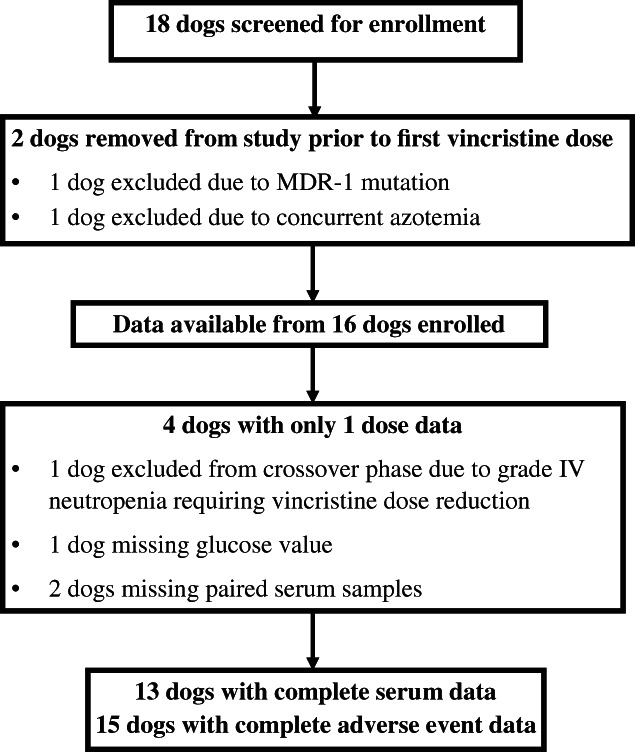
Flow chart provides details of dog screening, inclusion and exclusion from study

Eighteen client‐owned dogs were enrolled. All dogs were diagnosed with multi‐centric lymphoma via cytology and intended to initiate treatment with the CHOP protocol. Dogs were treated with cyclophosphamide (250 mg/m^2^ PO rounded to the nearest 25 mg capsule) and furosemide (1‐2 mg/kg PO) 7 to 10 days prior to enrolment with no reported GI, constitutive or haematological effects. Dogs were treated with prednisone pre‐enrolment (0.5‐1 mg/kg) and continued on the same dose throughout the study period. Seven patients were tested for *MDR‐1* gene mutation (*ABCB1*) through the Washington State University, and homozygous normal dogs were enrolled (n = 6). Two dogs were removed from the study following enrollment and prior to vincristine administration. One because of an *MDR‐1* mutation and one because of elevated renal values at initial screening. One dog (dog 8) was removed from the study because of a grade IV neutropenia following the first vincristine dose. This dog's AE data were included within the first dose analysis. Fifteen dogs completed the study period. Two dogs (dog 9 and dog 10) were removed from the serum ELISA testing because of inadequate sample availability and one dog (dog 2) only had glucose concentration reported from the fasted dose. Thus, 15 dogs were included in the AE analysis and 13 were included in the serum analysis. Figure 1 and Table 1 outline dog specific details to summarize the study population, which consisted of mainly large breed neutered dogs. Five dogs were fasted for their first vincristine treatment, and 10 dogs were fed for their first treatment. The majority of dogs (12/15) were fed twice daily as part of their normal husbandry, two dogs (dogs 4 and 17) could not be confirmed as free fed or fed twice daily, one dog (dog 16) was normally free fed. Thus, fasting duration was approximately 34 hours for most dogs. Dogs were distributed unequally because of the coin flip method of randomization, however order of treatment was not found to be significant for any of the variables evaluated.

**TABLE 1:** Patient characteristics of dogs fasted prior to first treatment compared with patients fed prior to first treatment

Characteristics		Fasted first	Fed first
Number		5	10
Age (years)	Median	8	10
	Range	4‐11	4‐14
Sex	FS	2	4
	MN	2	7
	M	1	0
Weight (kg)	Median	31.1	26.7
	Range	27.7‐39.5	6.2‐44.5
Breed	Labrador Retriever	2	2
	Australian Shepherd	2	0
	Mixed	0	3
	Other	1	6

The protocol was well tolerated with mild GI, lethargy and myelosuppression AEs observed, typical of the expected vincristine AE profile. No AEs were attributed to the fasting protocol, as fasted dogs' GI AEs did not occur during the period of fasting, but occurred several days later, consistent with chemotherapy‐associated AEs. Anti‐nausea and anti‐diarrhoea therapeutic intervention were not necessary, as no dogs experienced greater than grade two GI AEs. However, one dog (dog 5) was progressively anaemic and was treated with gastroprotectants throughout the study protocol.

A significant decrease in nausea was noted when dogs were fasted prior to treatment with one dog (7%) experiencing nausea when fasted and eight dogs (53%) experiencing nausea when fed (*P* = .016). When evaluating the incidence of anorexia, a significant decrease was observed in fasted vs fed dogs (7% vs 60%; *P* = .008). The individual dog experiencing nausea and anorexia when fasted, experienced the same toxicity scores when fed. Lethargy was noted in 6 fasted dogs and 11 dogs when fed (40% vs 73%; *P* = .016). None of the dogs experienced vomiting episodes when fasted prior to treatment while three dogs vomited (grade 1) when fed prior to treatment, however, this difference was not significant (*P* = .25). Diarrhoea AEs were not significantly different between groups, with two dogs experiencing grade 1 diarrhoea when fasted, and three dogs experiencing grade 1 diarrhoea when fed (*P* = 1.0).

When evaluating an individual dog's complete GI and constitutional AEs profile (shown in Tables 2 and 3), one dog experienced no side effects throughout the protocol and four dogs (26%) experienced only one mild AE throughout both treatments. Individual's complete GI and constitutional AE profiles could not be evaluated statistically because of low incidence of AE. Interestingly, 13 dogs (87%) experienced improvement in at least one category when fasted prior to treatment, with 8 (53%) experiencing improvement in more than two categories. Dogs 5, 7, 10, 11, 13, and 16 experienced notable improvement in their GI and constitutional AEs profiles when fasted compared with when each dog was fed normally prior to treatment (Tables 2 and 3). Only one dog experienced an AE when fasted that did not occur when the dog was fed prior to treatment (dog 12; grade 1 diarrhoea).

**TABLE 2:** Adverse event (AE) grade profile of dogs fasted prior to first vincristine dose. Both normal feeding (Fed) and fasting (Fast) AE are reported for each dog

Dog		Vomiting	Nausea	Anorexia	Diarrhoea	Lethargy
5	Fed	1	1	1	1	1
	Fast	0	0	0	1	1
7	Fed	0	0	1	1	1
	Fast	0	0	0	0	0
8	Fed	X	X	X	X	X
	Fast	0	0	0	0	6
14	Fed	0	1	1	0	0
	Fast	0	0	0	0	0
16	Fed	0	1	1	0	1
	Fast	0	0	0	0	0
17	Fed	0	0	0	0	1
	Fast	0	0	0	0	0

**TABLE 3:** Adverse event (AE) grade profile of dogs fed prior to first vincristine dose. Both normal feeding (Fed) and fasting (Fast) AE are reported for each dog

Dog		Vomiting	Nausea	Anorexia	Diarrhoea	Lethargy
1	Fed	1	2	2	0	1
	Fast	0	2	2	0	1
2	Fed	0	0	0	0	0
	Fast	0	0	0	0	0
4	Fed	1	1	1	1	1
	Fast	0	0	0	0	1
6	Fed	0	0	0	0	1
	Fast	0	0	0	0	0
9	Fed	0	0	0	0	1
	Fast	0	0	0	0	1
10	Fed	0	1	1	0	2
	Fast	0	0	0	0	1
11	Fed	0	1	1	0	0
	Fast	0	0	0	0	0
12	Fed	0	0	0	0	0
	Fast	0	0	0	1	0
13	Fed	0	2	2	0	2
	Fast	0	0	0	0	1
15	Fed	0	0	0	0	1
	Fast	0	0	0	0	0

*Note*: “X” represents no data collected, as this dog was removed from the study following the fed dose.

Fasting and treatment order (fasted vs fed first) did not have a significant impact on neutrophil count. Similar instances of neutropenia were seen when dogs were fed vs fasted prior to treatment (4 neutropenic events in each group). Interestingly, when dogs were fed prior to treatment there was trend of increasing severity of neutropenia, with 2 dogs experiencing a grade 2 neutropenia causing treatment delay while all neutropenia events noted within the fasted group were grade 1. Two dogs experienced mild thrombocytopenia when fed and none experiencing thrombocytopenia when fasted.

**FIGURE 2:**
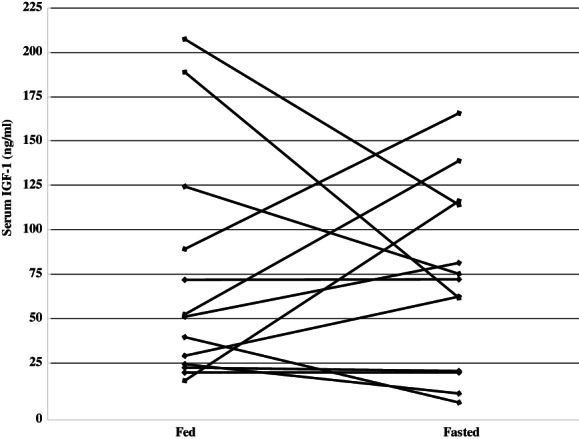
Insulin‐like growth factor‐1 (IGF‐1) serum concentrations. Serum IGF‐1 was measured via ELISA prior to each vincristine treatment. Each line represents an individual dog's IGF‐1 concentration when fasted vs fed normally

Serum glucose remained within normal limits with no statistical difference between fasted or fed time points. Fasted patients had significantly reduced serum insulin levels compared with fed patients (median when fasted: 73 ng/L [range: 39‐212 ng/L]); median when fed: 102 ng/L [range: 25‐485 ng/L]; *P* = .01). Serum IGF‐1 concentration was not significantly impacted by fasting (Figure 2).

## 5 DISCUSSION

This prospective crossover clinical trial evaluated the effect of fasting on vincristine‐associated GI, constitutive and haematological AEs, as well as and serum glucose, IGF‐1 and insulin levels. Our results demonstrate that fasting for 24 hours prior to treatment with vincristine significantly reduced the incidence of nausea, anorexia and lethargy, and significantly decreased serum insulin. In contrast, fasting prior to vincristine treatment did not significantly impact vomiting, diarrhoea, neutrophil count, or serum glucose or IGF‐1 concentration.

There is growing evidence that fasting reduces the GI effects of chemotherapy in animal and human patients. Previous reports indicate decreased vomiting associated with doxorubicin when dogs are fasted, and decreased GI AEs associated with a variety of chemotherapy agents when human patients voluntarily fasted.^7^, ^8^ In a prospective, randomized, crossover study of 34 women with gynaecological cancer, patients were randomized to eat normally or fast for three cycles of chemotherapy. The patients reported improved QOL and reduced fatigue when fasting during treatment, and the majority (31 of 34 patients) elected to fast for their subsequent treatments.^21^ Similarly, in our study population, several owners elected to continue the fasting protocol for the remainder of their pets' treatments because of the observed benefit.

There are multiple proposed mechanisms to explain the GI protective effect of fasting. Fasting causes reduced GI cellular proliferation through G1 cell cycle blockade, and therefore these cells may be less sensitive to chemotherapeutics, which preferentially target actively dividing cells.^22^, ^23^, ^24^ Withers et al, hypothesized GI cells in the G1 phase may be less sensitive to doxorubicin, which preferentially targets cells in S phase.^7^, ^25^ Similarly, vincristine primarily targets cells in M phase and thus G1 cell cycle blockade may be protective.^23^ in vitro studies in acute lymphoblastic leukaemia cells reported G1 cells were more susceptible to vincristine; however, a follow‐up study found this effect limited to high doses and was not repeatable with another cell line.^26^, ^27^ Another proposed mechanism is that nutrient deprivation from fasting triggers various cellular pathways to invest energy in repair and maintenance rather than proliferation. This is supported by a study where mice fasted for 24 hours prior to a high dose of etoposide had less DNA damage noted in small intestinal stem cells 3 hours after treatment compared with the mice fed normally, despite similar levels of DNA damage between groups had been noted 1.5 hours after treatment. Thus, DNA repair is hypothesized to be more efficient during periods of nutrient deprivation.^15^

The most investigated potential regulator of cellular stress resistance during fasting is IGF‐1. Mice with low serum IGF‐1 because of gene deletion are relatively resistant to high dose chemotherapy; this benefit was negated when IGF‐1 is administered IV.^10^ Serum IGF‐1 has been shown to decrease in fasted states, however, robust reduction in humans requires 48 hours of fasting.^28^, ^29^, ^30^ Fasting causes low serum insulin levels, which leads to growth hormone resistance in the liver and consequently inhibits hepatic IGF‐1 production. Thus, one would expect glucose, insulin and IGF‐1 to be positively correlated. In our study, we noted significantly reduced insulin when dogs were fasted; however, this was not associated with IGF‐1 reduction. Dogs with lymphoma may have abnormal carbohydrate metabolism, as this group has been reported to have higher insulin levels compared with controls.^31^, ^32^ However, a recent study found no difference in serum insulin, IGF‐1 or glucose in dogs with lymphoma compared with controls.^33^ These parameters were not correlated in our study population, although based on previous reports, it is possible that longer fasting intervals would be necessary to observe such serum concentration changes.^21^, ^28^, ^29^, ^30^

Withers et al reported significantly reduced delayed‐type vomiting in dogs fasted for approximately 18 hours prior to doxorubicin treatment and 6 hours following treatment with no significant difference in other AE or IGF‐1 compared with fed dogs.^7^ Similarly, we found no reduction in IGF‐1 to explain a significantly altered GI and constitutive AE profile.

Additionally, we report a significant difference in multiple GI AEs. More robust improvement in the incidence of GI AEs may be because of a longer duration of fasting in our study (approximately 28 hours prior to treatment and 6 hours following treatment) and the relatively rapid terminal half‐life of vincristine compared with doxorubicin (47.2 minutes vs 20 hours, respectively).^20^, ^34^ Refeeding after a period of nutrient scarcity causes enhanced mucosal proliferation, which peaks around 24 hours after replenishment, and thus protective effects of fasting may be diminished if the chemotherapeutic is present within tissue longer.^22^

Fasting may also reduce the myelosuppressive effects of chemotherapy through IGF‐1 mediated cellular resistance and enhanced DNA repair mechanisms of circulating haematopoietic cells and those within the bone marrow, similar to modification of GI AEs.^10^, ^15^ Additionally, fasting may promote haematopoietic stem cell protection as bone marrow collected from fasted mice treated with cyclophosphamide revealed significantly reduced levels of cyclophosphamide‐induced apoptosis in all haematopoietic stem cells, particularly short‐term haematopoietic stem cells and multi‐potent progenitors compared with mice fed ad libitum.^35^ As further support of bone marrow AE's attenuation through fasting, researchers conducting a prospective trial in breast cancer patients fed or fasted prior to chemotherapy found no significant difference in IGF‐1 levels; however, mean erythrocyte and thrombocyte levels significantly higher in the fasted group (7 days post‐treatment).^16^ DNA repair of circulating cells was hypothesized to be more efficient in the fasted group because of significantly higher levels of double‐strand breaks in circulating lymphocytes in the non‐fasted group (7 days post‐chemotherapy). There was no significant difference in neutrophil counts between groups 7 days post‐chemotherapy, although all patients were treated with granulocyte‐stimulating factor analogue.^16^ Interestingly, other in vivo studies evaluating the effect of fasting on chemotherapy haematological AEs has found no significant difference.^7^, ^8^ In our study population, there was not a significant difference in neutrophil count between fasted or fed groups; however, we noted a trend of increasing severity of neutropenia when dogs were fed prior to treatment.

There are several important limitations of this study. The study was not blinded and relied heavily on client reporting of constitutional and GI AEs, and compliance with the timing of fasting. Thus, there is potential for bias in reporting subjective AEs such as nausea and lethargy. No owners reported accidentally feeding their dog during the fasting period, however, this cannot be guaranteed as dogs were not housed in‐hospital. Additionally, groups were unequally distributed because of the coin flip method of randomization, causing the majority of dogs to be fasted prior to their second dose of vincristine. This study used a lower vincristine dose than described in other reports because of the authors' experience treating dogs at Oregon State University.^36^, ^37^ However, dogs served as their own control, and all dogs were treated with the same dose of vincristine (0.6 mg/m^2^). Further study would be needed to assess the effect of fasting with a higher dose of vincristine (0.7 mg/m^2^). Additionally, all dogs had received cyclophosphamide prior to enrolling in the study and receiving their first dose of vincristine. Cyclophosphamide causes dose‐dependent myelosuppression 8 to 10 days post administration and GI toxicity is rare at standard veterinary doses (200‐250 mg/m^2^).^38^, ^39^ High doses (500‐650 mg/m^2^) used prior to bone marrow transplantation uncommonly causes GI toxicity within 48 hours of administration.^39^ Dogs had at least a 7‐day washout from cyclophosphamide prior to starting the study; however, delayed AEs from cyclophosphamide may have influenced AE profiles attributed to each vincristine dose. Additionally, this study excluded small dogs (<5 kg) because of concerns for potential hypoglycemia associated with fasting. As such, the patient population consisted of mostly large breed (>25 kg) neutered dogs, and conclusions from this study should only be made for medium to large‐size dogs. Small breed dogs have increased mass‐specific metabolic rates compared with larger dogs and fasting may have a more profound effect on serum glucose levels.^40^ Further evaluation of the safety and efficacy of fasting in cancer‐bearing small dogs is warranted. It is important to note that this study was not designed to assess the effects of fasting related to differences in patient cancer diagnosis, stage of disease or immunophenotype of lymphoma.

All dogs enrolled were diagnosed with lymphoma, and the majority were B‐cell. It is possible the effect of fasting on AE associated with vincristine, and glucose, insulin and IGF‐1 concentrations vary with patient stage and immunophenotype of lymphoma.

Finally, dogs were prescribed prednisone throughout the study period, which may have affected our results. However, dogs were required to have tolerated this medication for at least 1 week and were maintained at the same dose throughout the trial period. As a comparison, a study of dogs diagnosed with inflammatory bowel disease found no significant effect of prednisone on IGF‐1.^41^

Interestingly in some dogs, we noted a wide range of IGF‐1 concentration between duplicate samples, and serum colour was grossly different between samples from fasted and fed time points. ALPCO ELISA serum insulin manufacturer claims to have no interference with triglyceride, bilirubin, and haemoglobin and IGF‐1 serum ELISA is reported to have no interference of triglyceride, bilirubin and haemoglobin at concentrations below 100 mg/mL, 200 ug/mL, or 1 mg/mL, respectively.^42^ Dogs with hyper‐bilirubinemia were not included in our study; however, we were unable to measure lipaemia or haemolysis, thus we concede that it is possible that haemolysis and/or lipaemia may have affected our values. Similar variance has not been reported in other studies, but it is not always noted if assays were run in duplicate or if results were presented as the mean.

## 6 CONCLUSION

Adverse effects related to chemotherapy remain a significant limitation to dose intensity, potential outcome as well as a cancer patient's QOL. Our findings suggest fasting is a reasonable treatment to consider for GI and constitutional AEs associated with vincristine in large breed dogs. Additional studies are warranted to validate the potential benefits of fasting prior to chemotherapy treatment in a larger cohort and explore optimal timing of re‐feeding.

## CONFLICT OF INTEREST

The authors declare no conflicts of interest.

## ETHICS STATEMENT

This clinical trial was registered with the American Veterinary Medical Association Animal Health Database (AAHSD000379) and approved by the Institutional Animal Care and Use Committee at Oregon State University (4804).

## Data Availability

The data that supports the findings of this study are available from the corresponding author upon reasonable request.
